# Extensive skin ulcerations in an HIV-positive patient with AIDS: An atypical giant acquired perforating dermatosis-chronic prurigo spectrum presentation

**DOI:** 10.1016/j.jdcr.2026.02.007

**Published:** 2026-02-11

**Authors:** Juris Podoļanskis, Kristīne Nevidovska, Oksana Mahmajeva, Kristīne Ābeltiņa, Lāsma Kalnbērza

**Affiliations:** a Department of HIV/AIDS (Dermatology-Venerology), Riga East University Hospital, Latvian Centre of Infectious Diseases, Riga, Latvia; b Department of Pathology, Riga East University Hospital, Centre of Pathology, Riga, Latvia; c Department of HIV/AIDS, Riga East University Hospital, Latvian Centre of Infectious Diseases, Riga, Latvia

**Keywords:** acquired perforating dermatosis, AIDS, chronic prurigo, dermatology, dermatopathology, HIV, prurigo nodularis, pruritus, skin ulcers

## Introduction

Four main perforating dermatoses exist: Kyrle disease, reactive perforating collagenosis, elastosis perforans serpiginosum, and perforating folliculitis.^1^ Acquired perforating dermatosis (APD) refers to pruritic perforating eruptions linked to systemic disease, showing transepidermal elimination of dermal components regardless of substrate, often with overlapping patterns.^1^, ^2^, ^3^ Rare “giant” variants present as lesions >1 cm, sometimes with keratotic plugs.^4^ Prurigo nodularis (PN) can overlap, but large ulcerations are uncommon.^2^^,^^5^

We report an unusual case of extensive, intensely pruritic ulcerations without central keratotic plugs in a patient newly diagnosed with HIV, who had initially been diagnosed with PN during the early evolution of the lesions before presenting to our dermatology-venerology department.

## Case report

**Fig 1:**
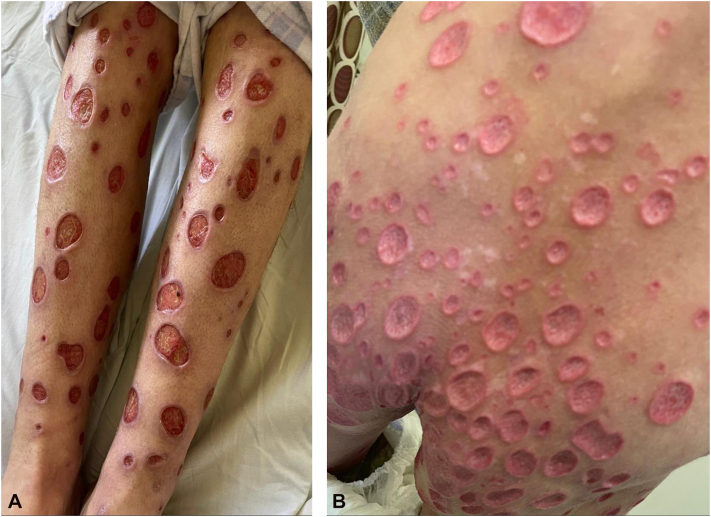
Clinical presentation of the patient’s lesions. Multiple ulcerative lesions on **(A)** both legs and **(B)** the lower back and gluteal region, varying in size (some >1 cm) and lacking keratotic plugging. Several lesions are coalescing.

A woman in her mid-40s presented with multiple bilateral crateriform ulcerations, many >1 cm with serous discharge, involving the lower extremities, gluteal region, lower back, and arms (Fig 1). Several lesions coalesced into larger ulcers, accompanied by severe pruritus and intermittent bleeding. The eruption began 1 year earlier as pruritic papules and nodules diagnosed as PN by prior clinicians, with ulceration developing approximately 1 month before admission. HIV testing was positive, prompting transfer to the HIV/AIDS unit for further evaluation (Table I). Imaging studies were nonspecific.

**Table I:** Laboratory analysis results

Test	Result	Reference range	Note
Lymphocytes (absolute)	0.37 × 10^3^/μL	1.00-3.50	Very low
Hemoglobin	9.40 g/dL	12.0-14.0	Low
MCV	88.4 fL	80-97	Normal
CRP	9.81 mg/L	0-5	Mildly elevated
Urea	6.76 mmol/L	2.76-8.07	Normal
Creatinine	42 μmol/L	44-80	Slightly low
eGFR	150 mL/min/1.73 m^2^	-	-
Total protein	55 g/L	66-87	Low
Albumin	27.4 g/L	35-52	Low
Glucose	5.40 mmol/L	4.11-5.89	Normal
Iron	7.14 μmol/L	5.8-34.5	Normal
Ferritin	172 ng/mL	13-150	High
Hepatitis B	Negative	-	Negative
Hepatitis C	Negative	-	Negative
Syphilis serology	Negative	-	Negative
CMV (DNA)	5190 copies/mL	-	Positive
HIV ½ (viral load)	5,580,000 copies/mL	-	Positive (HIV-1)
CD4^+^ (absolute)	0.000 × 10^9^/L	0.400-1.300	Very Low
ANA/ENA panel	Normal	-	Normal
IgE	20740 IU/mL	<100	High
TSH	2.8502 μIU/mL	0.35-4.94	Normal
FT4	0.92 ng/dL	0.70-1.48	Normal
*Pneumocystis jirovecii* (nasopharyngeal swab)	Negative	-	Negative
*Toxoplasma gondii* IgG	Negative	<8 IU/mL negative, >11 IU/mL positive	Negative
Tuberculosis (DNA/QuantiFERON)	Negative	-	Negative
*Aspergillus* (blood)	Negative	-	Negative
*Cryptococcus* antigen (blood)	Negative	-	Negative
*Cryptococcus neoformans* (blood/pharyngeal)	Negative	-	Negative
Blood culture	No growth	-	Negative
Ulcer swab culture	Pseudomonas aeruginosa growth	-	Positive
Urine culture	Pseudomonas aeruginosa & Escherichia coli growth	-	Positive

**Fig 2:**
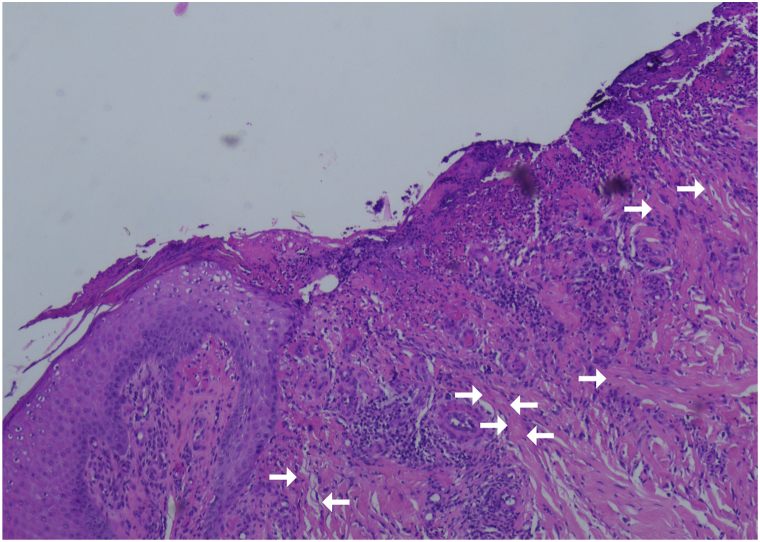
Histologic findings of an ulcerated lesion (H&E stain, 10× magnification). Granulation tissue shows dense lympho-leucocytic and macrophage infiltration. Vertically oriented collagen fibers (*white arrows*) are visible. Minor artifacts are present but do not affect interpretation.

**Fig 3:**
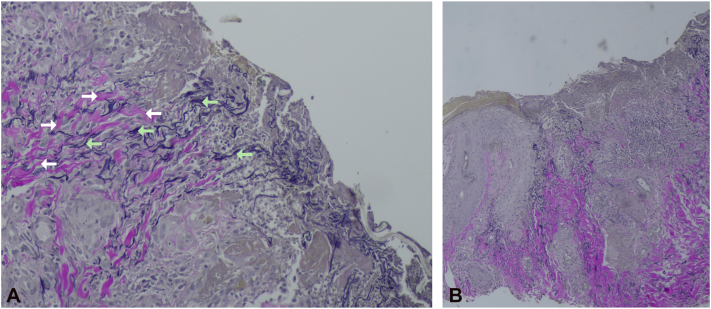
Histologic findings of an ulcerated lesion (Van Gieson stain, **(A)** 10× and **(B)** 4× magnification). Altered, vertically oriented collagen (*white arrows*) and elastic fibers (*bright green arrows*) are visible within and around the granulation tissue.

Four punch biopsies from ulcerated lesions showed epidermal ulceration, mixed inflammation, and reactive epidermal changes, initially favoring ulcerated PN (Fig 2). Given reported comparable cases, Van Gieson staining was performed and revealed vertically oriented, altered collagen and elastic fibers within granulation tissue; however, definitive transepidermal elimination through intact epidermis or well-formed channels was not identified (Fig 3). Alternative diagnoses-including pyoderma gangrenosum, ecthyma gangrenosum, fungal or viral infections (including CMV), neoplasia, and granulomatous dermatoses-were excluded clinically and histopathologically.

Considering the clinical presentation, histopathologic findings, and available literature, we interpret this case as an atypical manifestation possibly along the spectrum of APD and chronic prurigo (PN) associated with HIV/AIDS.

**Fig 4:**
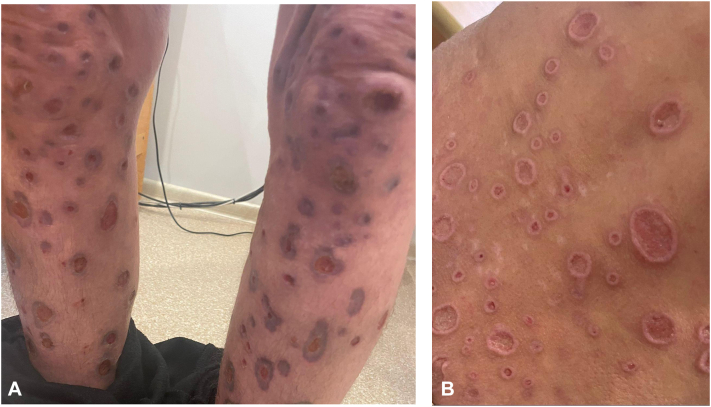
Clinical presentation of the patient’s lesions at 1-month follow-up after initiation of ART and supportive therapy. Lesions on **(A)** the legs and **(B)** the lower back show signs of remission.

After HIV/AIDS confirmation, ART with bictegravir/emtricitabine/tenofovir alafenamide (50/200/25 mg daily) was initiated, together with clarithromycin and trimethoprim-sulfamethoxazole. Ulcers were irrigated with purified water containing 0.1% betaine and polyhexanide, followed by topical steroid–fusidic acid to surrounding skin. The patient was discharged for outpatient follow-up. After 1 month, marked clinical improvement was observed with ART, systemic antibiotics, topical therapy, reduced pruritus, and decreased scratching (Fig 4).

## Discussion

No universally accepted diagnostic criteria exist for APD.^1^^,^^6^ Early definitions include: (1) transepidermal elimination of dermal components, (2) the specific substance being eliminated (collagen, elastic fibers, or keratin), (3) umbilicated papulonodular lesions with central keratin plugging, and (4) onset after 18 years of age.^6^^,^^7^

Pruritus is common in HIV,^8^ and both APD and PN are pruritic dermatoses affecting areas subjected to chronic scratching.^1^^,^^7^ While PN typically presents with papulonodular lesions, extensive ulcerative variants are uncommon.^5^ In APD, intense pruritus, scratching, and koebnerization suggests that mechanical trauma may contribute to lesion development.^1^^,^^7^

We identified 2 cases resembling ours. Satoshi and Kazutoshi Murao reported a diabetic patient with ulcerative pruritic lesions lacking central crusting or keratin plugs; no biopsy was performed, and the case was interpreted as prurigo nodularis, with APD considered in the differential.^5^ Naik et al described a comparable presentation as an atypical giant APD with perforating collagenosis-like changes.^4^ Similarly, our patient exhibited crateriform lesions >1 cm, a feature reported in “giant” variants with or without keratotic plugging and distinct from classic papulonodular APD. Collectively, these cases highlight ulcerative, prurigo-like presentations that challenge traditional APD criteria.

APD is most commonly associated with diabetes mellitus (DM) and chronic renal failure (CRF), and less frequently with HIV/AIDS and other systemic conditions.^1^^,^^3^ Proposed mechanisms in DM and CRF include vasculopathy, microdeposits, and metabolic alterations.^1^^,^^3^ PN has likewise been linked to metabolic and endocrine disorders, including DM and dialysis-dependent CRF, highlighting overlap between these entities.^9^ Our patient had no evidence of DM or CRF. Reported “giant” APD cases predominantly occur in patients with systemic disease similar to classic APD.^10^ In advanced HIV/AIDS, profound immune dysfunction may contribute to exaggerated tissue injury and impaired repair, producing extensive ulcerative lesions. IL-1-mediated activation of matrix metalloproteinases has been implicated in APD pathogenesis,^1^ and IL-1β-driven upregulation of MMP-1 in HIV/AIDS may offer a speculative mechanistic link.^2^^,^^11^^,^^12^ PN also demonstrates increased MMP expression, including MMP-1.^13^ Further studies are needed to clarify pathogenic pathways in HIV/AIDS and atypical ulcerative presentations.

Histopathologically, “true” APD has been described as showing transepidermal elimination of altered dermal components via discrete epidermal channels or focal defects, whereas PN usually displays pseudoperforation phenomena without elimination or alteration of dermal matrix structures.^2^ In our case, classic transepidermal elimination through intact epidermis-a defining histopathologic feature of acquired perforating dermatosis-could not be conclusively demonstrated. Additionally, certain histologic features of PN can resemble those traditionally attributed to APD, further complicating the distinction between the 2 entities.^14^^,^^15^ Some literature suggests that APD may represent a subtype of PN, highlighting a potential spectrum.^14^^,^^15^ Therefore, the histopathologic findings in this case are not diagnostic in isolation and must be interpreted cautiously in conjunction with the clinical presentation, lesion evolution, and exclusion of alternative causes.

Clinical improvement following hospitalization most likely reflects initiation of antiretroviral therapy, improvement in systemic immune status, reduction in pruritus, and decreased mechanical trauma from scratching.

APD and chronic prurigo (PN) represent overlapping entities in the differential diagnosis of pruritic papulonodular eruptions, including atypical ulcerative variants such as in our case. In practice, distinguishing between APD and PN often has limited impact on management, as both are treated with pruritus control, lesion care, and evaluation for underlying systemic disease. The key clinical relevance lies in recognizing atypical or ulcerative presentations, supporting a spectrum-based interpretation rather than a strict dichotomy and prompting exclusion of alternative ulcerative dermatoses. The “giant” designation is therefore best viewed as a descriptive marker of disease extent.

Limitations include the absence of molecular or immunologic studies and reliance on limited published data, particularly in HIV/AIDS. The “giant” APD variant is rare; one comparable report lacked histopathologic confirmation,^5^ while another showed perforating collagenosis-like changes,^4^ limiting direct comparison. Our case expands the literature by demonstrating an ulcerative, prurigo-like presentation with perforating features in a severely immunocompromised patient, supporting a perforating-prurigo spectrum rather than a discrete APD subtype. Histopathologic findings may aid diagnosis but should be interpreted cautiously given increasing recognition of overlap with prurigo nodularis.

## Conflicts of interest

None disclosed.
